# Influence of benzene exposure, fat content, and their interactions on erythroid-related hematologic parameters in petrochemical workers: a cross-sectional study

**DOI:** 10.1186/s12889-020-08493-z

**Published:** 2020-03-23

**Authors:** Xue Zhang, Qifei Deng, Zhini He, Jie Li, Xiaoju Ma, Zhaorui Zhang, Dehua Wu, Xiumei Xing, Jing Peng, Hongyu Guo, Ming Huang, Liping Chen, Shanfeng Dang, Yanqun Zhu, Zhengbao Zhang, Boyi Yang, Hailan Wang, Wen Chen, Yongmei Xiao

**Affiliations:** 1 Department of Occupational and Environmental Health, School of Public Health, Sun Yat-sen University, 74 Zhongshan 2nd Rd, Guangzhou, 510080 Guangdong China; 2 Guangdong Provincial Key Laboratory of Food, Nutrition and Health, School of Public Health, Sun Yat-sen University, Guangzhou, 510080 Guangdong China; 3 Food Safety and Health Research Center, School of Public Health, Southern Medical University, Guangzhou, 510515 Guangdong China; 4 Division of Vital Statistics and Death Surveillance, National Center for Chronic and Noncommunicable Disease Control and Prevention, Chinese Center for Disease Control and Prevention, Beijing, 100050 China; 5 Guangdong Provincial Key Laboratory of Occupational Disease Prevention and Treatment, Guangdong Province Hospital for Occupational Disease Prevention and Treatment, Guangzhou, 510300 Guangdong China; 6 Occupational Disease Prevention and Treatment Institute of Sinopec Maoming Petrochemical Company, Maoming, 525000 Guangdong China

**Keywords:** Benzene exposure, Fat content, Interactions, Erythroid-related hematologic parameters

## Abstract

**Background:**

Ubiquitously distributed benzene is a known hematotoxin. Increasing evidence has suggested that erythroid-related hematologic parameters may be sensitive to benzene exposure. Fat content, which is also closely associated with erythroid-related hematologic parameters, may affect the distribution and/or metabolism of benzene, and eventually benzene-induced toxicity.

**Methods:**

To explore the influence of benzene exposure, fat content, and their interactions on erythroid-related hematologic parameters, we recruited 1669 petrochemical workers and measured their urinary S-phenylmercapturic acid (SPMA) concentration and erythroid-related hematological parameters. Indices for fat content included body fat percentage (BF%), plasma total cholesterol (TC) and triglycerides (TG), and occurrence of fatty liver.

**Results:**

The dose-response curve revealed U-shaped nonlinear relationships of SPMA with hematocrit (HCT) and mean corpuscular hemoglobin concentration (MCHC) (*P*-overall < 0.001, and *P*-nonlinear < 0.015), as well as positive linear associations and r-shaped nonlinear relationships of continuous fat content indices with erythroid-related hematological parameters (*P*-overall ≤0.005). We also observed modification effects of fat content on the associations between benzene exposure and erythroid-related hematological parameters, with workers of lower or higher BF% and TG more sensitive to benzene-induced elevation of MCHC (*P*_interaction_ = 0.021) and benzene-induced decrease of HCT (*P*_interaction_ = 0.050), respectively. We also found that some erythroid-related hematologic parameters differed between subgroups of workers with different SPMA levels and fat content combination.

**Conclusions:**

Our study suggested that benzene exposure, fat content, and their interactions may affect erythroid-related hematological parameters in petrochemical workers in a complex manner that are worthy of further investigation.

## Background

Benzene is a representative single-ring aromatic compound which ubiquitously distributes in our environment due to its high volatility and extensive emission sources. Benzene occurs naturally in crude oil and gas emission from volcanoes or forest fires. However, most environmental contamination and human exposure results from man-made sources like the emissions from motor vehicles, cigarette smoke, and the production and usage of petroleum products and common consumer goods, such as adhesives, paints, rubber products, solvents, and pharmaceutical products [1]. With the increasing development of industrialization and globalization, many developing countries have achieved great growth in the production of petrochemical products. This expands the population of petrochemical workers potentially exposed to benzene [2]. Furthermore, occupational exposures are likely to occur at higher concentrations than those encountered in the general environment.

Benzene is a serious public health concern and has been classified as a known human carcinogen [3]. After entering the body mostly through inhalation, benzene is metabolically activated by cytochrome P450 enzymes, and its metabolites distributes to lipid-rich tissues (including bone marrow) according to their fat content and rate of perfusion by blood, resulting in multiple adverse health effects. Hematotoxicity is the most widely-studied health effect for benzene exposure [4]. Multiple studies have linked benzene exposure with the abnormality of hematologic parameters, such as the reduction in the counts of white blood cell (WBC), red blood cell (RBC), neutrophil, and lymphocyte, even at low exposure levels (< 1 ppm) [5–7]. A decreased WBC count has been considered as a key clinical sign of benzene-induced hematotoxicity [8]. However, increasing evidence supports that erythrocytes may be more sensitive at low benzene exposure levels. A large-scale study conducted in Korean workers revealed that RBC count was the only hematologic parameter that was reduced by low-level benzene exposure (< 1 ppm) in male workers [9]. Our previous studies had also shown that RBC count was decreased in C57BL/6 J male mice treated with 1 ppm benzene, and that erythroid cell differentiation of hematopoietic progenitor cells was inhibited by exposure to low-dose hydroquinone (a toxic metabolite of benzene) [10, 11]. However, the literature investigating the effects of benzene exposure on erythroid-related hematologic parameters contains inconsistent findings, something needing resolution through the conduct of more epidemiologic studies.

As benzene mainly accumulates in the fat-enriched tissues upon entry into human body [12], fat content in the body and in tissues or organs (such as blood and liver) might affect the distribution and/or metabolism of benzene, and may eventually affect benzene-induced toxicity. Moreover, studies have demonstrated that fat content may also have an effect on erythroid-related hematologic parameters. The percentage of erythroid precursors of bone marrow cells was significantly increased in rats fed with a fatty-rich diet [13]. High-fat diet induced dysfunction of erythrocytes by increasing their oxidative stress levels and reducing their survival in a rat model of high fat diet-induced obesity [14]. Also, patients with non-alcoholic fatty liver disease were more likely to have a high red cell distribution width (RDW) and elevated hemoglobin (Hb) levels [15, 16]. In addition, indicators for fat content in blood, such as serum cholesterol (TC) and triglycerides (TG), were also associated with hematological diseases [17]. However, few studies have investigated the effects of fat content on the associations of benzene exposure with erythroid-related hematologic parameters.

Thus, in order to investigate the influence of benzene exposure, fat content in the body, in blood, and in liver, and their interactions on erythroid-related hematologic parameters, we performed a cross-sectional study among 1669 petrochemical workers. We quantified their benzene exposure levels by measuring a widely-used internal exposure marker, S-phenylmercapturic acid (SPMA), estimated their body fat percentage (BF%), measured TC and TG in blood, evaluated the occurrence of fatty liver by ultrasound, and measured multiple commonly-used erythroid-related hematologic parameters. We used restricted cubic spline (RCS) functions to characterize the dose-response associations of benzene exposure and fat content with erythroid-related hematologic parameters. We further probed into their interaction effects on erythroid-related hematologic parameters. The present study might further enhance our knowledge about the determinants of the severity of benzene induced-erythroid hematotoxicity.

## Methods

### Study design and setting

In order to investigate the influence of benzene exposure, fat content, and their interactions on erythroid-related hematologic parameters, we conducted a cross-sectional study in workers from two state-run petrochemical companies which are located in Guangzhou and Maoming in Guangdong Province, China.

### Study participants

We firstly recruited a total of 1802 workers and then intentionally excluded: a) workers who had been working for less than 1 year in their petrochemical plants; b) workers with self-reported and/or diagnosed carcinomas, hematological diseases, and/or immune diseases; c) workers taking any medicine in the preceding 2 weeks; d) workers experiencing X-ray examination for any reason in the preceding 1 month; and e) workers unwilling to provide biological samples or doing so in insufficient volume. Eventually, a total of 1669 workers were recruited in this study.

After participants signed informed consent, we administered a structured questionnaire to collect their information on demographic characteristics, lifestyle (such as smoking and drinking habits), medical history, and occupational experience (such as working years and workplaces). Participants who had smoked ≥1 cigarette/day for ≥1 year were considered as smokers, and those who drank wine and/or other alcoholic beverages at least once a week for ≥1 year were classified as drinkers. Workers that had been working in workshops such as petroleum refining, chemical production, and petroleum processing were considered as benzene-exposed group, while administration staffs were considered as non-exposed subjects.

After the interview, we collected ~ 20 mL morning urine sample and ~ 2 mL ethylenediaminetetraacetic acid (EDTA)-anticoagulated venous blood sample from each participant after overnight fasting. The present study had received ethical approval from the Ethical Review Committee at School of Public Health, Sun Yat-Sen University.

### Measurement of urinary SPMA concentration

Urinary SPMA concentration was determined with liquid chromatography/electrospray tandem mass spectrometry (LC-MS/MS) described in detail previously [18]. In brief, 5 mL urine sample for each participant was centrifuged at 800 *g* for 5 min. An aliquot of 500 μL supernatant was mixed with 500 μL of 10 mM sodium acetate buffer (pH = 6.3), and treated with 50 μL of 10 μg/L SPMA-d5 working solution. After solid-phase extraction (Waters Oasis® MAX), the samples were analyzed with LC-MS/MS (Agilent, US). SPMA (purity 98%) is used for qualitative analysis, and standard curves were drawn for quantitative analysis. SPMA-d5 working solution as internal standard was used for quality control. The inter-assay coefficient of variation (CV) (10 different sample preparations, 10 measurements at different days by different persons) was 10.21%. The detection limit for urinary SPMA was 0.01 μg/L and the concentrations of samples with levels below detection limit were substituted to 0.005 μg/L. SPMA concentration was standardized by urinary creatinine and expressed as μg/g creatinine.

### Measurement of fat content indices

We measured the height of our participants using a wall-mounted stadiometer. The participants stood upright on a firm surface, looking straight ahead, arms at sides, with shoes removed and feet together. Their shoulders, buttocks, and heels were required to be touching the wall. We also measured the weight for participants with a calibrated standing balance scale. Participants removed shoes, heavy outer clothing, and items from pockets. Height and weight were measured twice for each participant to ensure accurate measurement. The average height and weight were used to calculate body mass index (BMI) according to the following formula:

$$ \mathrm{BMI}=\mathrm{Weight}\ \left(\mathrm{kg}\right)/\mathrm{Height}\ {\left(\mathrm{m}\right)}^2 $$

Then, we calculated BF% for each participant according to the following formula [19]:

$$ \mathrm{BF}\%=\left(1.20\times \mathrm{BMI}\right)+\left(0.23\times \mathrm{Age}\right)-10.8\times \mathrm{Gender}-5.4 $$

In this formula, “Gender” =1 for male workers, while “Gender” =0 for female workers.

Plasma samples extracted from EDTA-anticoagulated blood were used for the quantitative analysis of TC and TG by automatic biochemical analyzer (Cobas, Switzerland). The inter-assay CVs for TC and TG (10 different sample preparations, 10 measurements at different days by different persons) were 8.62 and 12.97%, respectively. Two professional sonographers separately detected the occurrence of fatty liver disease for each participant by abdominal ultrasound examination based on clinical diagnostic criteria of fatty liver [20], and the concordance rate was 100%.

### Measurement of erythroid-related hematologic parameters

In the present study, we used automatic hematology analyzer (Sysmex, Japan) to measure seven commonly-used erythroid-related hematologic parameters in EDTA-anticoagulated venous blood samples, including RBC count (× 10^12^/L), Hb concentration (g/L), hematocrit (HCT) (%), mean corpuscular volume (MCV) (fL), mean corpuscular hemoglobin concentration (MCHC) (g/L), RDW-coefficient of variation (RDW-CV) (%), and RDW-standard deviation (RDW-SD) (fL). These hematologic parameters were measured within 2 h after sample collection, and each blood sample was assayed in duplicate. The average levels for these erythroid-related hematologic parameters were included in the following statistical analyses.

### Statistical analyses

We examined the distributions of all numerical variables by Kolmogorov-Smirnov tests. As the urinary SPMA concentration had a left-skewed distribution, it was normalized by natural logarithm (ln) transformation before statistical analyses [21]. Several variables were adjusted as confounders in our statistical analyses, including age (continuous), gender (male/female), smoking status (smokers/non-smokers), drinking status (drinkers/non-drinkers), working years (continuous), and workplace (exposed/control group). As BF% was calculated based on age and gender, we didn’t adjust age and/or gender in all following analyses involving BF%.

We evaluated the differences of general characteristics between the exposed workers and controls with Student’s *t*-test for continuous variables and chi-square test for categorical variables. We analyzed the between-group differences of SPMA concentration, fat content indices, and erythroid-related hematologic parameters by multivariate analysis of covariance, with adjustment for the above-mentioned confounding variables except for workplace.

RCS models, which have been widely-used to represent nonlinear relationships for continuous independent variables, were used to characterize patterns of changes in erythroid-related hematologic parameters with the changes in continuous exposure (including SPMA, BF%, TC, and TG) in the total population, while adjusting for the above-mentioned covariates. Continuous exposure was included into RCS models with 4 default knots located at the 5th, 35th, 65th, and 95th percentiles. The reference values on RCS curves were set at the median values for continuous exposure, and the values on Y axis represented the differences in erythroid-related hematologic parameters between individuals with any value of continuous exposure with those with median levels. The outputted *P*-overall indicate *P* values for test of overall association, and *P*-nonlinear indicate *P* values for test of nonlinear association. We used covariate-adjusted generalized linear models (GLMs) to analyze the effects of occurrence of fatty liver disease (no =0, and yes = 1) on erythroid-related hematologic parameters (as dependent variables) in the total population, and those of SPMA and all fat content indices with dependent variables in workers with different general characteristics. We further explored the modification effects of general characteristics on these associations by adding an interaction term of independent variable (continuous) and stratified variable (categorical) in GLMs.

Then, we divided our workers into three subgroups (T1, T2, and T3) according to the tertiles of BF%, TC, and TG, respectively, and divided workers into two subgroups according to the occurrence of fatty liver (No/Yes). We analyzed the associations of SPMA with erythroid-related hematologic parameters in subgroups with different fat content indices by covariate-adjusted GLMs, and explored the modification effects of fat content indices on these associations by adding an interaction term of SPMA (continuous) and fat content categories (categorical) in GLMs. In order to evaluate the combined effects of benzene exposure and fat content, we also divided our participants into four subgroups based on the median SPMA concentration (low/high) and the median continuous fat content indices or the occurrence of fatty liver. Workers with low SPMA and with low BF%, TC, TG, or without fatty liver were defined as the reference groups, and the differences of erythroid-related hematologic parameters between other subgroups and controls were evaluated with covariate-adjusted GLMs.

RCS analyses were performed by rms package in R software (Version 3.5.2), and other statistical analyses were performed using SPSS (Version 22.0). Two-tailed *P* < 0.05 was considered statistically significant.

## Results

### Subject characteristics

The general characteristics, benzene exposure levels, fat content indices, and erythroid-related hematologic parameters among workers in different workplaces were presented in Table 1. Significant differences were observed in terms of all general characteristics between controls and exposed workers (all *P <* 0.05). Median SPMA levels in the exposed workers were about 2.1-fold higher than that in control subjects (*P* < 0.001). BF% and MCHC were significantly lower while HCT, MCV, and RDW-SD were higher in exposed workers than control subjects (all *P* < 0.05).

**Table 1:** Characteristics, benzene exposure levels, fat content, and erythroid-related hematologic parameters in workers in different workplaces

Variables^a^	Control group (*n* = 338)	Exposed group (*n* = 1331)	*P* value
**General characteristics**			
Age (years)	46.46 ± 7.25	40.20 ± 7.37	< 0.001^b^
Gender (male/female, %male)	237/101 (70.10)	1014/317 (76.20)	0.022^c^
Smoking status (smoker/non-smoker, %smoker)	98/240 (29.00)	466/865 (35.00)	0.037^c^
Drinking status (drinker/non-drinker, %drinker)	103/235 (30.50)	490/841 (36.80)	0.030^c^
Work years (years)	25.94 ± 8.45	18.93 ± 8.52	< 0.001^b^
**Benzene exposure levels**			
SPMA (μg/g creatinine)	0.18 (0.09, 0.44)	0.37 (0.15, 1.09)	< 0.001^d^
**Fat content indices**			
BF% (%)	26.66 ± 5.29	23.67 ± 23.67	0.003^e^
TC (mmol/L)	5.48 ± 0.94	5.20 ± 0.96	0.410^d^
TG (mmol/L)	1.66 ± 1.11	1.59 ± 1.41	0.305^d^
Fatty liver (yes/no, %yes)	84/250 (25.10)	311/1016 (23.40)	0.551^c^
**Erythroid-related hematologic parameters**			
RBC count (×10^12^/L)	4.94 ± 0.58	4.96 ± 0.58	0.157^d^
Hb (g/L)	142.71 ± 13.93	143.60 ± 14.53	0.286^d^
HCT (%)	42.62 ± 3.52	43.25 ± 4.07	0.027^d^
MCV (fL)	86.90 ± 8.15	87.70 ± 8.17	0.030^d^
MCHC (g/L)	336.87 ± 8.22	331.92 ± 10.53	< 0.001^d^
RDW-CV (%)	12.90 ± 1.03	13.17 ± 1.36	0.792^d^
RDW-SD (fL)	39.87 ± 2.57	43.86 ± 3.35	< 0.001^d^

*Abbreviations*: *SPMA* S-phenylmercapturic acid, *BF%* Body fat percentage, *TC* Plasma total cholesterol, *TG* Plasma triglycerides, *RBC* Red blood cell, *Hb* Hemoglobin concentration, *HCT* Hematocrit, *MCV* Mean corpuscular volume, *MCHC* Mean corpuscular hemoglobin concentration, *RDW-CV* Red cell distribution width-coefficient of variation, *RDW-SD* Red cell distribution width-standard deviation

^a^ Values were shown by n (%), mean ± SD, or median (25th percentile, 75th percentile)

^b^ Student’s *t*-test

^c^ Chi-squared test

^d^ Multivariate analysis of covariance, adjusted by age, gender, smoking status, drinking status, and work years

^e^ Multivariate analysis of covariance, adjusted by smoking status, drinking status, and work years

### Associations of SPMA and fat content indices with erythroid-related hematologic parameters

**Fig. 1:**
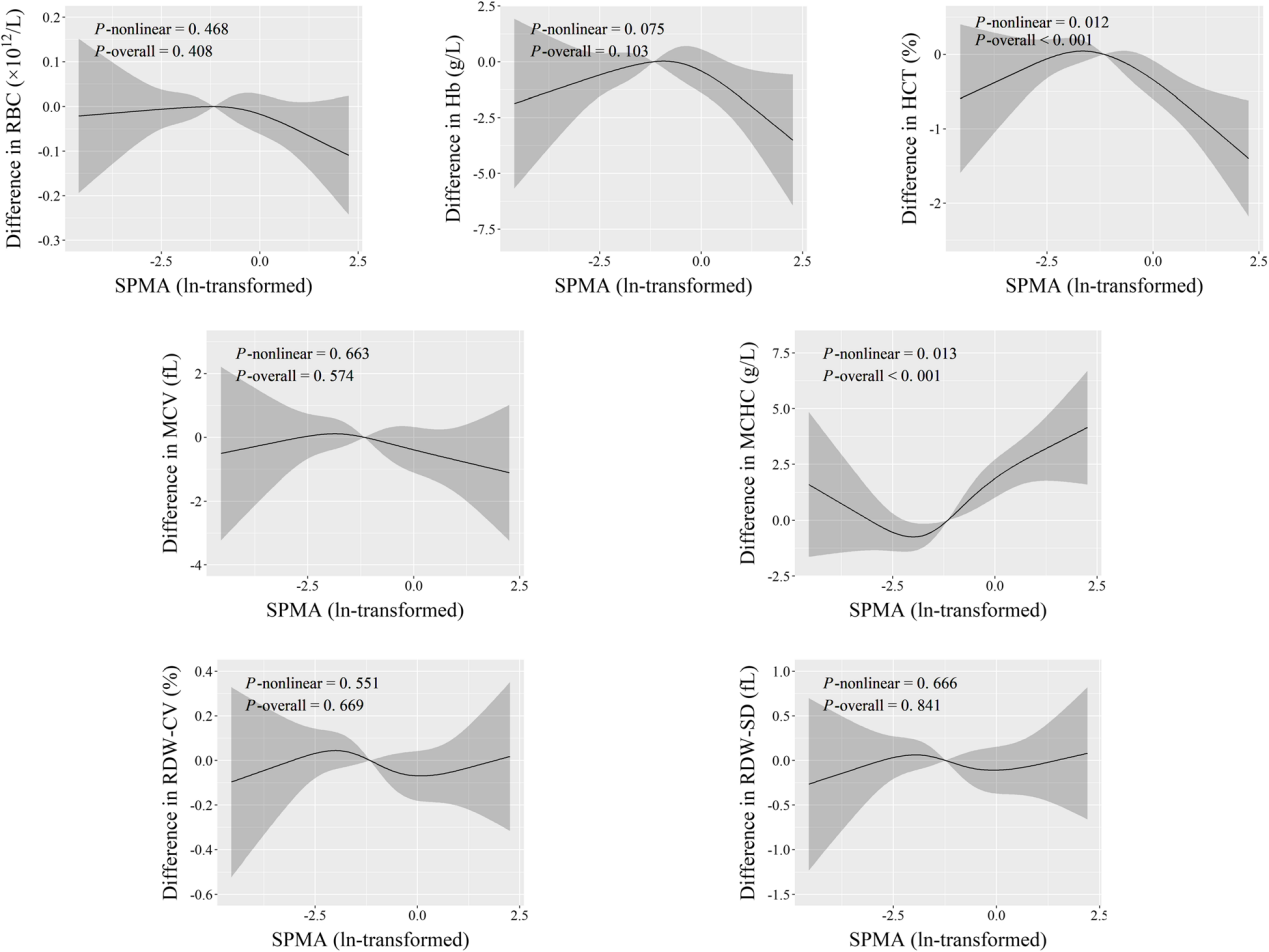
Adjusted dose-response associations of SPMA with erythroid-related hematologic parameters

**Fig. 2:**
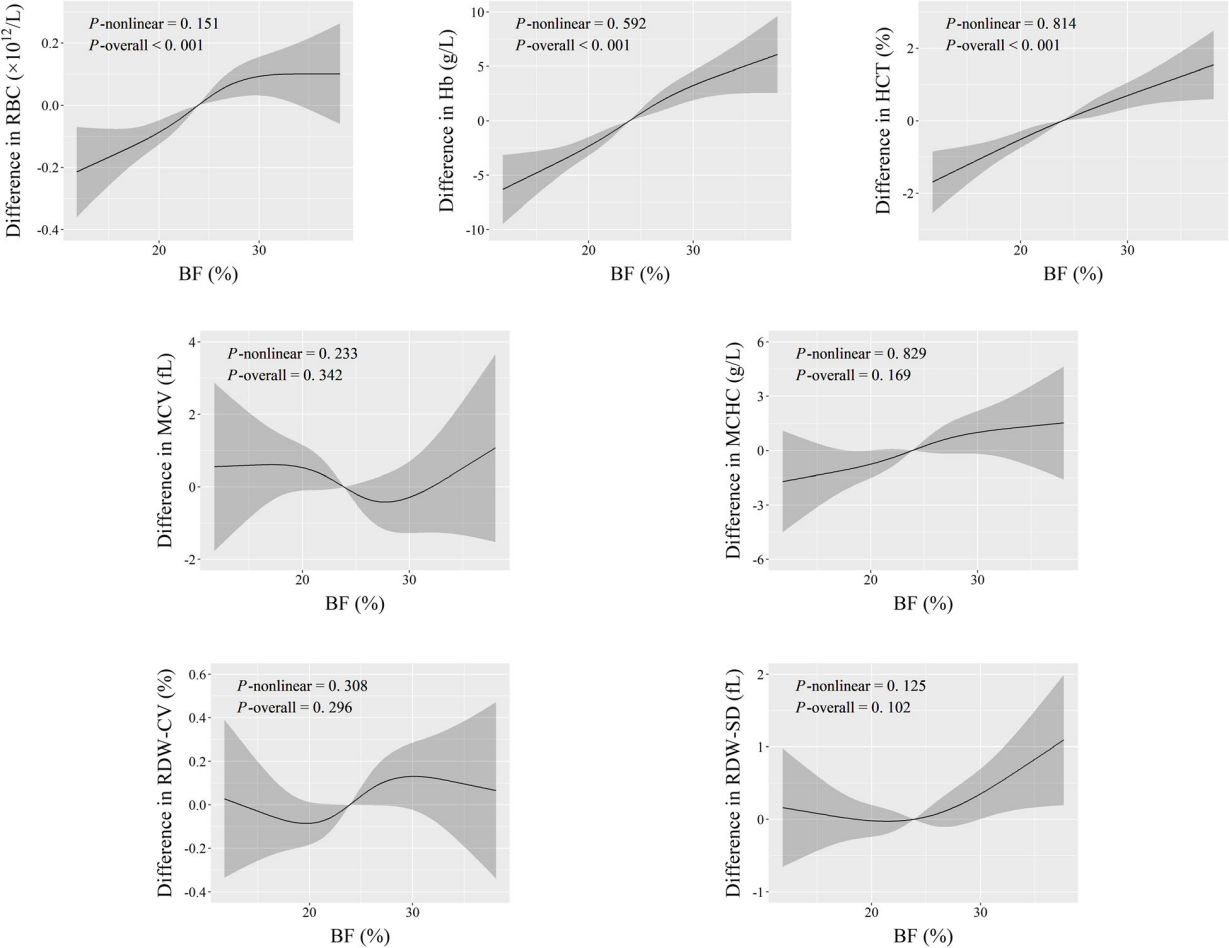
Adjusted dose-response associations of BF% with erythroid-related hematologic parameters

**Fig. 3:**
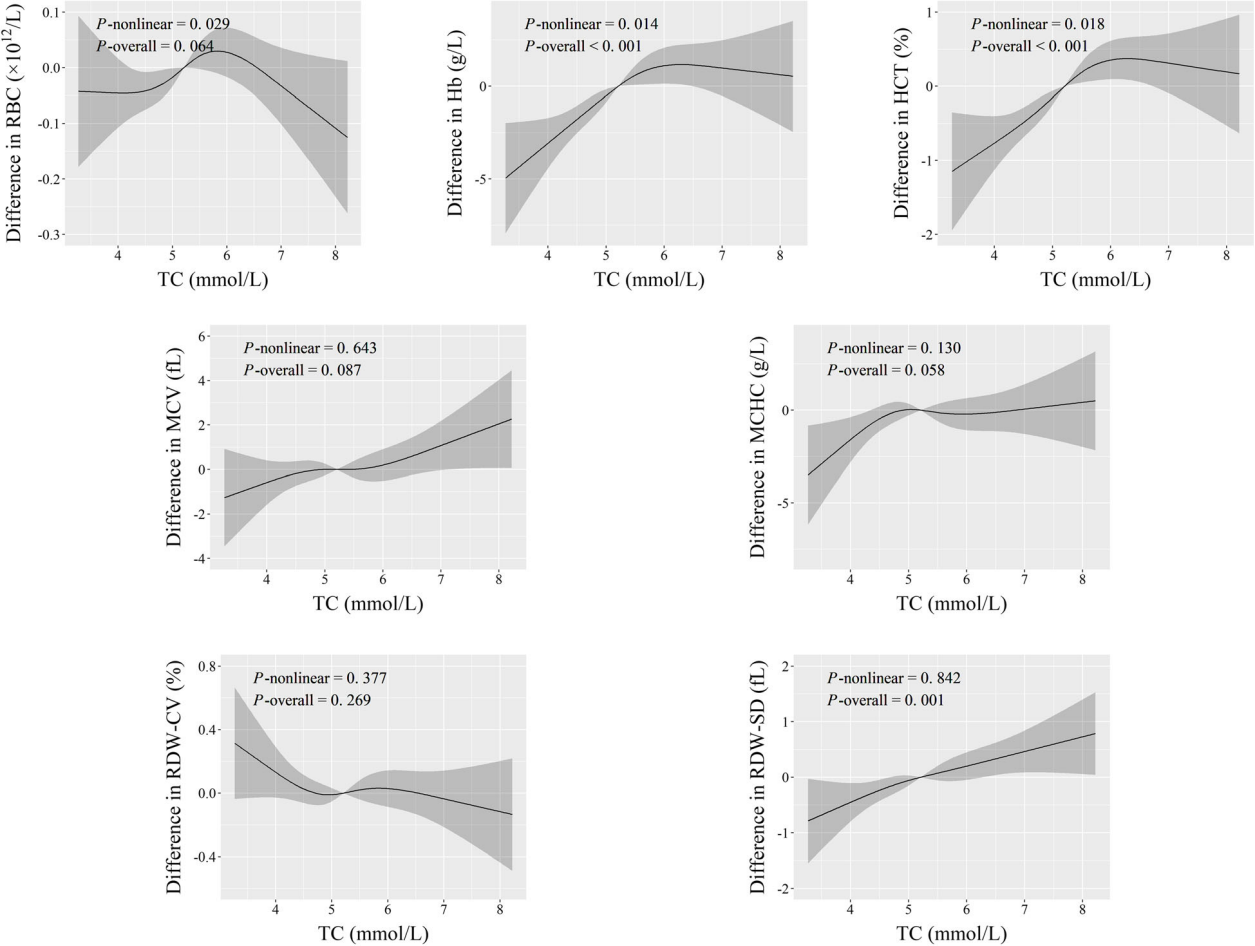
Adjusted dose-response associations of TC with erythroid-related hematologic parameters

**Fig. 4:**
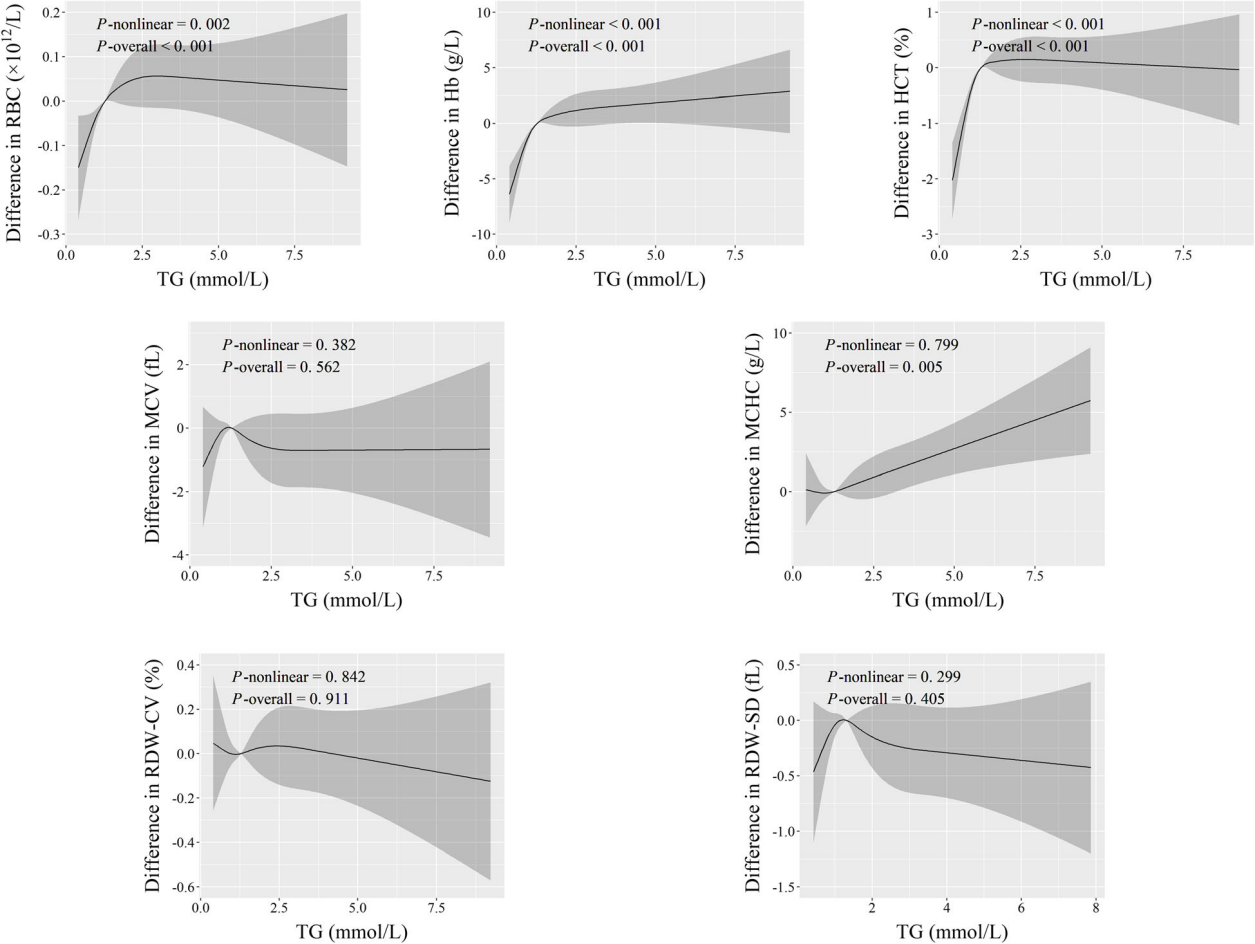
Adjusted dose-response associations of TG with erythroid-related hematologic parameters

We conducted RCS analyses to characterize the dose-response relationships of SPMA and continuous fat content indices with erythroid-related hematologic parameters (Figs. 1, 2, 3 and 4). We observed that SPMA had inverse U-shape and U-shape nonlinear relationships with HCT and MCHC, respectively (*P*-overall < 0.001, and *P*-nonlinear < 0.015) (Fig. 1). When ln-transformed SPMA was lower than about − 1.8, HCT increased and MCHC decreased with the increase of SPMA. However, when ln-transformed SPMA was higher than about − 1.8, HCT decreased and MCHC increased with an increase of SPMA.

As for continuous fat content indices, the dose-response curves revealed positive linear associations of BF% with RBC count, Hb, and HCT, of TC with RDW-SD, and of TG with MCHC (*P*-overall ≤0.005, and *P*-nonlinear > 0.05) (Figs. 2, 3 and 4). Additionally, both TC and TG had r-shape nonlinear relationships with Hb and HCT (*P*-overall < 0.001, and *P*-nonlinear < 0.02). TG also had r-shape nonlinear dose-response relationship with RBC count (*P*-overall < 0.001, and *P*-nonlinear = 0.002). Hb and HCT increased until around 6 mmol/L of TC and then slightly declined. RBC count, Hb, and HCT increased until around 1.25 mmol/L of TG and then levels off (Figs. 3 and 4).

Similarly, workers with fatty liver disease had relatively higher RBC count, Hb, and HCT (*P* < 5.0 × 10^− 5^) (Table 2). These results, taken together with those in Figs. 1, 2, 3 and 4, suggest that benzene exposure and fat content might have complex effects on erythroid-related hematologic parameters.

**Table 2:** Erythroid-related hematologic parameters in workers with and without fatty liver disease

Erythroid-related hematologic parameters	Without fatty liver (*n* = 1266)	With fatty liver (*n* = 395)	*P* value^a^
RBC count (×10^12^/L)	4.91 ± 0.58	5.13 ± 0.55	3.60 × 10^−5^
Hb (g/L)	141.78 ± 14.66	148.88 ± 11.96	2.24 × 10^−8^
HCT (%)	42.67 ± 4.07	44.68 ± 3.19	4.46 × 10^− 7^
MCV (fL)	87.54 ± 8.24	87.66 ± 7.86	0.338
MCHC (g/L)	332.66 ± 10.50	333.43 ± 9.76	0.102
RDW-CV (%)	13.13 ± 1.39	13.08 ± 1.05	0.543
RDW-SD (fL)	42.94 ± 3.59	43.49 ± 3.48	0.853

^a^ GLMs with adjustment for age, gender, smoking status, drinking status, work years, and workplace

In addition, we also investigated these associations in workers with different characteristics (including gender, smoking status, and/or drinking status) with GLMs (See Tables S1-S5, Additional file 1). These results indicated that general characteristics could modify the associations of SPMA and fat content with some erythroid-related hematologic parameters, with such influence more pronounced in females, non-smokers, and non-drinkers (*P*_interaction_ < 0.05).

### Interactions and combined effects of SPMA and fat content on erythroid-related hematologic parameters

**Fig. 5:**
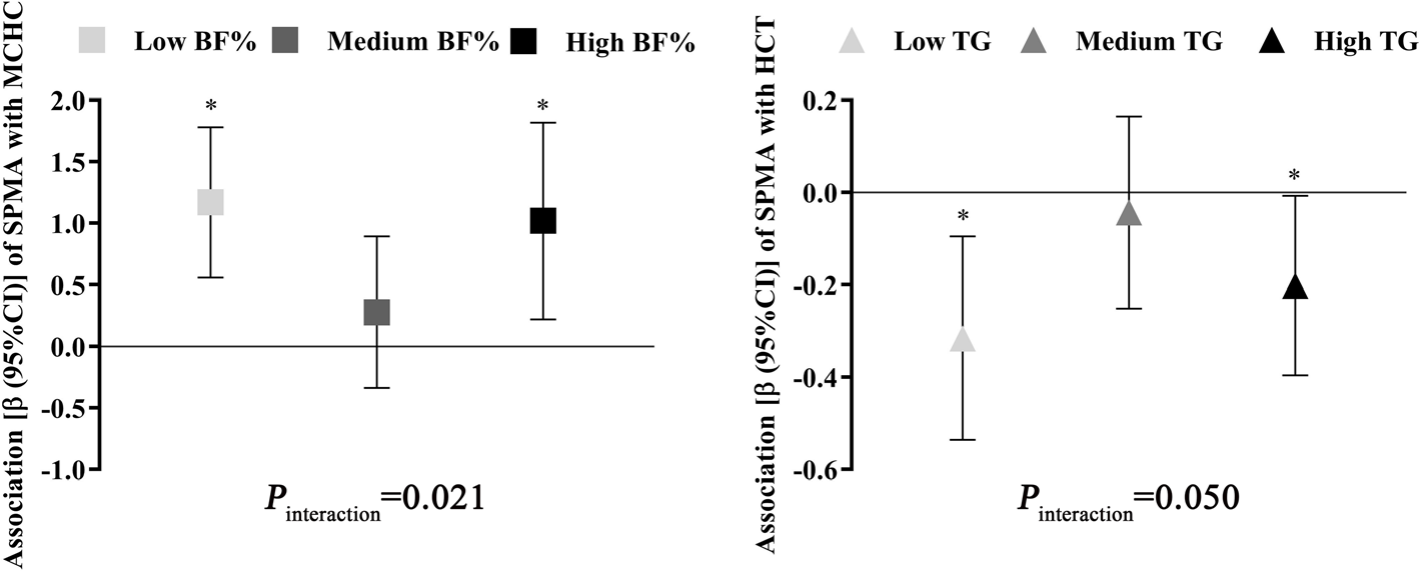
Significant modification effects of fat content on the associations between SPMA and erythroid-related hematologic parameters. The lines represent the associations (β, 95% CI), and *P* values for interaction effects are calculated by modeling an interaction term of SPMA and fat content in GLMs with adjustment for age, gender, smoking status, drinking status, work years, and workplace. ^*^P_trend_ < 0.05, by GLMs adjusted for age, gender, smoking status, drinking status, work years, and workplace

To investigate whether benzene exposure and fat content have interaction effects on erythroid-related hematologic parameters, we analyzed the modification effects of fat content indices on the associations between urinary SPMA levels and erythroid-related hematological parameters. We found that both T1 and T3 subgroups of BF% had a more pronounced positive association of SPMA with MCHC, while the association was weaker and insignificant in workers of T2 group (*P*_interaction_ = 0.021). Similarly, we also observed that both T1 and T3 subgroups of TG also had a more pronounced negative association of SPMA with HCT, while the association was also weaker and insignificant in workers of T2 group (*P*_interaction_ = 0.050) (Fig. 5 and Table S6, Additional file 1). Associations of SPMA with other erythroid-related hematological parameters were not significantly different among workers with different fat content levels (*P*_interaction_ > 0.050) (Table S6, Additional file 1).

We also investigated the combined effects of benzene exposure with fat content on erythroid-related hematologic parameters. We divided our participants into four subgroups according to their median level of SPMA and median levels of continuous fat content indices or the occurrence of fatty liver, with workers of “Low SPMA + Low fat indices or without fatty liver” as the reference groups (Table 3). The general observation is that RBC count, Hb, and HCT was significantly decreased in workers with higher BF%, and significantly elevated in workers with higher TC and TG and with fatty liver, regardless of SPMA levels (between-group *P* < 0.05). MCV was also significantly elevated in workers with higher TC, once again without respect to SPMA levels (between-group *P* < 0.05). MCHC and RDW-SD values did not significantly differ between the four subgroups (between-group *P* > 0.05). RDW-CV, however, was slightly, but consistently elevated in workers with higher SPMA and lower BF%, TC, TG, or without fatty liver, and decreased in workers with higher SPMA and higher TC and TG or with fatty liver (between-group *P* < 0.05).

**Table 3:** Comparison in erythroid-related hematologic parameters among workers with different benzene exposure levels and fat content

Subgroups	RBC count (×10^12^/L)	Hb (g/L)	HCT (%)	MCV (fL)	MCHC (g/L)	RDW-CV (%)	RDW-SD (fL)
Low SPMA+ Low BF%	5.11 ± 0.52	147.01 ± 11.11	44.35 ± 2.86	87.53 ± 8.23	332.82 ± 9.29	13.03 ± 1.23	42.53 ± 3.36
Low SPMA + High BF%	4.81 ± 0.60*	138.75 ± 15.96**	41.76 ± 4.16*	87.35 ± 7.84	332.16 ± 10.83	13.19 ± 1.36	42.65 ± 3.45
High SPMA + Low BF%	5.06 ± 0.56	147.41 ± 11.37	44.20 ± 3.24	88.09 ± 8.26	333.79 ± 10.11	13.09 ± 1.22*	43.55 ± 3.72
High SPMA + High BF%	4.88 ± 0.60*	140.44 ± 16.56**	42.26 ± 4.74*	87.13 ± 8.42	332.38 ± 11.03	13.19 ± 1.42*	43.61 ± 3.72
Low SPMA+ Low TC	4.94 ± 0.57	141.82 ± 14.58	42.76 ± 3.86	87.13 ± 8.39	332.33 ± 10.29	13.20 ± 1.37	42.28 ± 3.24
Low SPMA + High TC	4.98 ± 0.57	143.81 ± 13.32	43.42 ± 3.47*	87.78 ± 7.50*	332.68 ± 9.67	13.10 ± 1.28	42.58 ± 3.39
High SPMA + Low TC	4.93 ± 0.63	141.43 ± 15.55	42.52 ± 4.48	86.92 ± 8.84	333.22 ± 11.81	13.21 ± 1.44*	42.90 ± 3.80
High SPMA + High TC	5.01 ± 0.54	146.43 ± 13.01*	43.87 ± 3.71	88.20 ± 7.75*	333.51 ± 9.60	13.10 ± 1.25**	44.01 ± 3.50
Low SPMA+ Low TG	4.90 ± 0.59	140.06 ± 13.41	42.35 ± 3.66	87.20 ± 8.77	331.81 ± 10.03	13.17 ± 1.41	42.34 ± 3.47
Low SPMA + High TG	5.03 ± 0.54	145.76 ± 14.08**	43.86 ± 3.57**	87.69 ± 7.04	333.24 ± 9.92	13.14 ± 1.24	42.52 ± 3.14
High SPMA + Low TG	4.83 ± 0.57	139.36 ± 15.18	41.83 ± 4.36	87.24 ± 8.55	332.97 ± 11.42	13.21 ± 1.53*	43.44 ± 3.64
High SPMA + High TG	5.10 ± 0.57	148.31 ± 12.42**	44.47 ± 3.52*	87.88 ± 8.12	333.73 ± 10.06	13.10 ± 1.15**	43.50 ± 3.73
Low SPMA+ Without fatty liver	4.91 ± 0.58	141.55 ± 14.31	42.71 ± 3.83	87.57 ± 8.16	332.07 ± 10.02	13.13 ± 1.38	42.54 ± 3.46
Low SPMA + With fatty liver	5.12 ± 0.53*	147.99 ± 13.15**	44.36 ± 3.37**	87.14 ± 7.43	333.92 ± 10.11	13.05 ± 1.03*	42.76 ± 3.16
High SPMA + Without fatty liver	4.91 ± 0.58	142.02 ± 15.09	42.64 ± 4.32	87.46 ± 8.37	333.15 ± 10.95	13.15 ± 1.40**	43.38 ± 3.69
High SPMA + With fatty liver	5.14 ± 0.56*	149.66 ± 10.78*	44.96 ± 3.02*	88.15 ± 8.23	332.98 ± 9.47	13.10 ± 1.07*	44.18 ± 3.63

Values were shown by mean ± SD

The between-group difference was calculated by GLMs with adjustment for age, gender, smoking status, drinking status, work years, and workplace

* *P* < 0.05, and ** *P* < 0.001when compared with the reference group

## Discussion

In the present cross-sectional study conducted in 1669 petrochemical workers, we found that benzene exposure and fat content in body, in blood, and in liver might affect erythroid-related hematologic parameters in linear, U-shaped, or r-shaped dose-response patterns. Furthermore, we found that BF% and TG might modify the associations of benzene exposure with erythroid-related hematologic parameters. Specifically, workers of low or high BF% were more sensitive to benzene-induced elevation of MCHC than their median BF% counterparts, and workers of low or high TG were more sensitive to benzene-induced decrease of HCT than their median TG counterparts. We also found that most erythroid-related hematologic parameters differed among subgroups with varying combinations of SPMA levels and fat content, indicating there were combined effects between benzene exposure and fat content on erythroid-related hematologic parameters.

The possible mechanisms underlying the benzene-mediated hematoxicity has been widely-studied in recent years [22]. Toxic benzene metabolites (such as hydroquinone, phenol, and catechol) produced by metabolism in the liver and bone marrow act on hematopoietic stem progenitor cells and mature blood cells, suppress the function of hematopoietic tissue, and cause abnormality in blood cell counts. Increasing evidence found that the erythrocyte is also sensitive to benzene exposure and even low-dose (< 1 ppm) benzene can disturb red blood cells [10, 11]. Thus, we focused on the associations of benzene with erythroid-related hematologic parameters in the present study. Although substantial previous studies have explored the linear relationship between benzene exposure and erythroid-related hematologic parameters [9, 23], their findings were inconsistent and few studies have further investigated whether there were any non-linear relationships. Since RCS allows easy visualization and flexible associations between an exposure and outcome, we applied this model to fully examine the dose-response relationships.

In the present study, urinary SPMA, a sensitive and specific benzene exposure biomarker which is rarely influenced by other factors (such as diet) [24], was found to have inverse U-shape and U-shape nonlinear relationships with HCT and MCHC, respectively, with the inflection points of ln-transformed SPMA at around − 1.8 (equivalent to about 0.17 μg/g creatinine of SPMA). HCT is the proportion of whole blood that is made up of red blood cells, and may be used to check for conditions such as anemia, dehydration, malnutrition, and leukemia. MCHC is the mean Hb concentration in a specific volume of red blood cells, and is calculated by dividing Hb by HCT. An increase in MCHC indicates autoimmune hemolytic anemia or hereditary spherocytosis, and a decrease indicates a thalassemia or an anemia due to iron deficiency. In the present study, the association of SPMA with MCHC was in the direction opposite to that of SPMA with HCT, namely with the increase of SPMA, HCT increased first and then decreased, while MCHC decreased first and then increased. This suggests that the relationship between benzene exposure and some erythroid-related hematologic parameters may be discrepant at different exposure levels. MCHC was found to be inversely associated with blood benzene in residents living near petrochemical areas at environmental exposure concentration [25], which was consistent with our finding that at lower benzene exposure levels, MCHC decreased with increasing SPMA. Furthermore, significantly decreased HCT and increased MCHC were observed in individuals working in high traffic and industrial areas with high concentration of benzene [26], which was also consistent with our finding that at higher benzene exposure levels, MCHC increased and HCT decreased with increasing SPMA. However, a study conducted in children observed negative correlation between HCT and benzene at environmental exposure levels [27], which is contrary to our result that HCT increased with increasing SPMA at lower exposure levels. This discrepancy might be explained by age-related differences in susceptibility to benzene, as toxicokinetic processes that determine dose differ between children and adults, not to mention vulnerability of the hematologic system to toxic insult. Taken together, our findings provided a new insight that benzene exposure could influence erythroid-related hematologic system in a nonlinear pattern which might be dependent on benzene exposure levels. Further studies are needed to clarify the exposure conditions under which the variable response of each erythroid-related hematologic parameter might occur.

Fat content is involved in metabolism and body energy reserves. Under-load or over-load of fat content may cause adverse health effects. Low fat content often reflects poor nutrition status and may increase susceptibility to diseases related to environmental pollutant exposure [28]. As for high fat content, it may cause inflammation and disturb blood cell counts [29], and is also an important risk factor for cardiovascular diseases and metabolic disorders [30]. In the present study, we used BF%, TC, TG, and the occurrence of fatty liver disease to represent fat content in body, in blood, and in liver. The reason for using BF% instead of BMI to represent the body fat content was that BF% is a better predictor for body fat mass based on age and gender [19, 31]. Studies have shown that some erythroid-related hematologic parameters, such as RBC count, Hb, and HCT, were significantly positively correlated with waist to hip ratio and waist circumference [32, 33]. In the present study, we also observed that BF%, TC, and TG are significantly positively associated with erythroid-related hematologic parameters in linear or r-shaped dose-response patterns, and workers with fatty liver disease had higher RBC count, Hb concentration, and HCT% than their non-diseased counterparts. The underlying mechanisms of the effects of fat content on erythroid-related hematologic parameters may hinge upon membranes of erythrocytes, which are rich in polyunsaturated fatty acids, being very sensitive to changes in fat content in vivo [34]. Excessive fat content may cause abnormal counts and function of RBCs by affecting their membrane composition, fluidity, and rheology [32].

We also conducted stratification analyses to understand the effect of general characteristics on the associations of benzene and fat content with erythroid-related hematologic parameters. Our findings indicated that most of these associations were more remarkable in females, in non-smokers, and in non-drinkers. Physiological differences between males and females may account for variable toxicological responses to poisons [35]. Studies have shown that females have a higher percentage of body fat, gender alters the biotransformation of benzene, and women may be more susceptible to blood alterations caused by benzene exposure [36]. Cigarette smoking is one of the most important sources of benzene, and studies have demonstrated that smoking may affect the associations of benzene exposure with blood cell counts [23]. In addition, smoking and drinking have been found to stimulate hematopoietic system, and result in the changes of hematologic parameters, such as RBC count, Hb, and MCV [37, 38]. Our findings and those of others suggest that more attention should be paid to the influence of gender and lifestyle factors (such as smoking and drinking) when surveilling the health of populations occupationally exposed to benzene.

Moreover, fat content might affect the distribution and/or metabolism of benzene, and eventually affect benzene-induced toxicity. However, few studies have investigated the interaction effects of benzene exposure with fat content on erythroid-related hematologic parameters. In the present study, we observed that the associations of SPMA with MCHC and HCT were significantly influenced by BF% and TG, respectively, with workers at the lower and higher extremes of each fat content index more sensitive to benzene-induced elevation of MCHC and decrease of HCT. These interesting findings indicated that lower and higher fat content may be risk factors for benzene-induced hematotoxicity. For example, a lower fat content might cause lower energic reserves and affect immunological function, impairing the ability to cope with environmental stressors [39]. On the other hand, chronic benzene exposure decreased white blood cell numbers, but only in rats that had a large volume of fat tissue [40]. Furthermore, we also found that most erythroid-related hematologic parameters differed among subgroups of workers having different combinations of benzene exposure and fat content (i.e., low or high BF%, TC, or TG and with or without fatty liver). As benzene mainly accumulates in lipid-rich tissue due to its lipophilicity [41], high fat content may allow for more benzene to accumulate in the body, slow the elimination of benzene and its active metabolites, and thus enhance the toxicity of benzene. Additionally, benzene exposure and high fat content can both induce excessive reactive oxygen species which might cause erythrocytic dysfunction and cell apoptosis, ultimately making RBCs more easily absorbed by spleen and inducing abnormal blood cell counts [42, 43]. Benzene exposure can also increase the production of proinflammatory cytokine [26], and obese individuals are considered to be in a low-grade inflammatory state [44]. Studies have indicated that cytokines and inflammatory environment might both interfere with the RBC maturation in bone marrow [45]. Moreover, some enzymes and polymorphisms in their encoding genes may affect both benzene-induced hematotoxicity and fat content levels. For example, polymorphisms in myeloperoxidase (MPO) (such as 463G > A) and NAD(P) H dehydrogenase quinone 1 (NQO1) (such as C609T) may alter enzyme activity, which is associated with TC levels [46, 47], TG levels [47], and the risk of benzene poisoning [48]. These enzymes and their genetic polymorphisms might be the underlying mechanisms of the interaction effects between benzene exposure and fat content on erythroid-related hematologic parameters. Although further studies are strongly needed to confirm our findings and explore underlying mechanisms for our observed hematologic changes, the present study strongly suggest fat content might influence the health status of benzene-exposed individuals. It remains less clear, however, under what circumstances a lower or higher fat content would increase one’s sensitivity to benzene-induced toxicity or prove protective.

Our study had some major strengths. Firstly, we used multiple fat content indictors to represent the fat content in body, in blood, and in liver. Furthermore, the present study was the first to examine the interaction effects of benzene exposure with fat content on erythroid-related hematologic parameters. Our findings may have important implications for the health surveillance of environmental and occupational benzene-exposed population alike, though considerable uncertainty exits as to whether our findings are extrapolatable to the general population. Finally, our subjects may be co-exposed to other hazards which might also influence erythroid-related hematologic parameters. Hence, further studies are needed to validate our findings, explore the potential mechanisms, and evaluate the effects of co-exposure to other hazards on our findings.

## Conclusions

In summary, the present study revealed benzene exposure and fat content in body, in blood, and in liver might all affect erythroid-related hematologic parameters. We also observed that fat content might affect the severity of benzene-induced erythroid-related hematotoxicity, with workers of lower or higher fat content more sensitive to benzene-induced erythroid-related hematotoxicity. We also found there might be combined effects between benzene exposure and fat content. Our findings may have some implications in health surveillance and prevention of benzene-induced erythroid-related hematotoxicity for environmental- and occupational-exposed populations. However, further studies should be performed to validate our current findings and explore their potential mechanisms.

## Supplementary information

**Additional file 1: Table S1.** Associations of urinary SPMA with erythroid-related hematologic parameters in different groups of gender, smoking status and drinking status. **Table S2.** Associations of BF% with erythroid-related hematologic parameters in different groups of smoking status and drinking status. **Table S3.** Associations of plasma TC levels with erythroid-related hematologic parameters in different groups of gender, smoking status and drinking status. **Table S4.** Associations of plasma TG levels with erythroid-related hematologic parameters in different groups of gender, smoking status and drinking status. **Table S5.** Associations of fatty liver occurrence with erythroid-related hematologic parameters in different groups of gender, smoking status and drinking status. **Table S6.** Associations of urinary SPMA with erythroid-related hematologic parameters in different fat content categories.

## Data Availability

The datasets used and/or analyzed during the current study are available from the corresponding author on reasonable request.

## Abbreviations

WBC: White blood cell
RBC: Red blood cell
RDW: Red cell distribution width
Hb: Hemoglobin
TC: Total cholesterol
TG: Triglycerides
SPMA: S-phenylmercapturic acid
BF%: Body fat percentage
RCS: Restricted cubic spline
EDTA: Ethylenediaminetetraacetic acid
LC-MS/MS: Liquid chromatography/electrospray tandem mass spectrometry
CV: Coefficient of variation
BMI: Body mass index
HCT: Hematocrit
MCV: Mean corpuscular volume
MCHC: Mean corpuscular hemoglobin concentration
RDW-CV: Red cell distribution width-coefficient of variation
RDW-SD: Red cell distribution width-standard deviation
ln-transformation: Natural logarithm transformation
*P*-overall: *P* values for test of overall association
*P*-nonlinear: *P* values for test of nonlinear association
GLMs: Generalized linear models
*P*_interaction_: *P* values for test of interaction
*P*_trend_: *P* values for test of linear association
MPO: Myeloperoxidase
NQO1: NAD(P) H dehydrogenase quinone 1
