# Supplementary material for: Influence of benzene exposure, fat content, and their interactions on erythroid-related hematologic parameters in petrochemical workers: a cross-sectional study
Source: BMC Public Health. 2020 Mar 23;20:382. doi: 10.1186/s12889-020-08493-z (PMC7092548; doi:10.1186/s12889-020-08493-z)
Supplement: Supplementary file 1 — Additional file 1: Table S1. Associations of urinary SPMA with erythroid-related hematologic parameters in different groups of gender, smoking status and drinking status. Table S2. Associations of BF% with erythroid-related hematologic parameters in different groups of smoking status and drinking status. Table S3. Associations of plasma TC levels with erythroid-related hematologic parameters in different groups of gender, smoking status and drinking status. Table S4. Associations of plasma TG levels with erythroid-related hematologic parameters in different groups of gender, smoking status and drinking status. Table S5. Associations of fatty liver occurrence with erythroid-related hematologic parameters in different groups of gender, smoking status and drinking status. Table S6. Associations of urinary SPMA with erythroid-related hematologic parameters in different fat content categories. [file 12889_2020_8493_MOESM1_ESM.doc]

**Additional File 1**

**Influence of benzene exposure, fat content, and** **their interactions on erythroid-related** **hematologic parameters in** **petrochemical workers: a cross-sectional study**

Xue Zhang^#^, Qifei Deng^#^, Zhini He, Jie Li, Xiaoju Ma, Zhaorui Zhang, Dehua Wu, Xiumei Xing, Jing Peng, Hongyu Guo. Ming Huang, Liping Chen, Shanfeng Dang, Yanqun Zhu, Zhengbao Zhang, Boyi Yang, Hailan Wang, Wen Chen, and Yongmei Xiao

^#^ These authors contributed equally to this work.

| **Table S1.** Associations of urinary SPMA with erythroid-related hematologic parameters in different groups of gender, smoking status and drinking status. | | | | | | | | | | | | | | |
| --- | --- | --- | --- | --- | --- | --- | --- | --- | --- | --- | --- | --- | --- | --- |
| Erythroid-related hematologic parameters | Gender | β (95%CI) | *P*_value_^a^ | *P*_interaction_^b^ |  | Smoking status | β (95%CI) | *P*_value_^a^ | *P*_interaction_^b^ |  | Drinking status | β (95%CI) | *P*_value_^a^ | *P*_interaction_^b^ |
| RBC count | Female | -0.003 (-0.043, 0.037) | 0.872 | 0.685 |  | Smokers | -0.018 (-0.051, 0.015) | 0.278 | 0.838 |  | Drinkers | -0.005 (-0.040, 0.030) | 0.780 | 0.867 |
|  | Male | -0.014 (-0.037, 0.010) | 0.251 |  |  | Non-smokers | -0.011 (-0.037, 0.015) | 0.394 |  |  | Non-drinkers | -0.012 (-0.037, 0.013) | 0.346 |  |
| Hb | Female | -0.130 (-1.248, 0.988) | 0.820 | 0.578 |  | Smokers | 0.260 (-0.403, 0.922) | 0.443 | 0.078 |  | Drinkers | 0.649 (-0.063, 1.360) | 0.074 | **0.013** |
|  | Male | -0.200 (-0.672, 0.272) | 0.405 |  |  | Non-smokers | -0.515 (-1.118, 0.089) | 0.095 |  |  | Non-drinkers | -0.565 (-1.133, 0.003) | 0.052 |  |
| HCT | Female | -0.210 (-0.501, 0.080) | 0.157 | 0.377 |  | Smokers | 0.015 (-0.165, 0.195) | 0.870 | **0.009** |  | Drinkers | 0.123 (-0.065, 0.311) | 0.200 | **0.006** |
|  | Male | -0.150 (-0.277, -0.022) | 0.022 |  |  | Non-smokers | **-0.292 (-0.451, -0.132)** | **3.49×10^-4^** |  |  | Non-drinkers | **-0.270 (-0.422, -0.119)** | **4.98×10^-4^** |  |
| MCV | Female | -0.555 (-1.290, 0.180) | 0.140 | 0.126 |  | Smokers | 0.291 (-0.236, 0.818) | 0.280 | **0.044** |  | Drinkers | 0.346 (-0.212, 0.905) | 0.225 | **0.013** |
|  | Male | -0.068 (-0.426, 0.291) | 0.711 |  |  | Non-smokers | **-0.431 (-0.848, -0.013)** | **0.043** |  |  | Non-drinkers | **-0.415 (-0.814, -0.015)** | **0.042** |  |
| MCHC | Female | 1.355 (0.332, 2.378) | 0.010 | 0.151 |  | Smokers | 0.658 (0.086, 1.231) | 0.025 | 0.349 |  | Drinkers | 0.390 (-0.218, 0.998) | 0.209 | 0.274 |
|  | Male | 0.720 (0.318, 1.123) | 4.61×10^-4^ |  |  | Non-smokers | 1.049 (0.524, 1.574) | 9.55×10^-5^ |  |  | Non-drinkers | 0.954 (0.456, 1.452) | 1.85×10^-4^ |  |
| RDW-CV | Female | -0.080 (-0.215, 0.056) | 0.251 | 0.194 |  | Smokers | 0.017 (-0.061, 0.096) | 0.665 | 0.290 |  | Drinkers | 0.011 (-0.077, 0.098) | 0.808 | 0.552 |
|  | Male | 0.003 (-0.050, 0.055) | 0.923 |  |  | Non-smokers | -0.042 (-0.109, 0.025) | 0.223 |  |  | Non-drinkers | -0.021 (-0.084, 0.041) | 0.502 |  |
| RDW-SD | Female | -0.134 (-0.409, 0.140) | 0.338 | 0.296 |  | Smokers | **0.231 (0.041, 0.421)** | **0.018** | **5.56×10^-4^** |  | Drinkers | 0.158 (-0.034, 0.351) | 0.107 | **0.007** |
|  | Male | 0.021 (-0.107, 0.148) | 0.750 |  |  | Non-smokers | **-0.192 (-0.339, -0.044)** | **0.011** |  |  | Non-drinkers | -0.102 (-0.247, 0.043) | 0.170 |  |
| ^a^ GLMs with adjustment for age, gender, smoking status, drinking status, work years, and workplace when appropriate.  ^b^ Adding an interaction term of SPMA and gender, smoking status, and drinking status, respectively, in GLMs with adjustment for age, gender, smoking status, drinking status, work years, workplace when appropriate. | | | | | | | | | | | | | | |

| **Table S2.** Associations of BF% with erythroid-related hematologic parameters in different groups of smoking status and drinking status. | | | | | | | | | |
| --- | --- | --- | --- | --- | --- | --- | --- | --- | --- |
| Erythroid-related hematologic parameters | Smoking status | β (95%CI) | *P*_value_^a^ | *P*_interaction_^b^ |  | Drinking status | β (95%CI) | *P*_value_^a^ | *P*_interaction_^b^ |
| RBC count | Smokers | **0.014 (0.002, 0.026)** | **0.018** | **0.004** |  | Drinkers | -0.007 (-0.018, 0.003) | 0.172 | **0.025** |
|  | Non-smokers | **-0.034 (-0.040, -0.028)** | **1.98×10^-28^** |  |  | Non-drinkers | **-0.030 (-0.036, -0.024)** | **8.51×10^-21^** |  |
| Hb | Smokers | **0.397 (0.165, 0.630)** | **8.65×10^-4^** | **3.51×10^-6^** |  | Drinkers | -0.095 (-0.323, 0.133) | 0.415 | **9.09×10^-5^** |
|  | Non-smokers | **-1.078 (-1.225, -0.930)** | **9.17×10^-43^** |  |  | Non-drinkers | **-0.990 (-1.140, -0.839)** | **1.59×10^-35^** |  |
| HCT | Smokers | **0.112 (0.050, 0.175)** | **4.31×10^-4^** | **1.83×10^-8^** |  | Drinkers | -0.051 (-0.111, 0.008) | 0.093 | **0.002** |
|  | Non-smokers | **-0.315 (-0.355, -0.275)** | **3.94×10^-49^** |  |  | Non-drinkers | **-0.280 (-0.321, -0.239)** | **7.64×10^-38^** |  |
| MCV | Smokers | -0.047 (-0.230, 0.137) | 0.618 | 0.143 |  | Drinkers | 0.022 (-0.140, 0.184) | 0.790 | 0.894 |
|  | Non-smokers | -0.050 (-0.138, 0.038) | 0.268 |  |  | Non-drinkers | -0.065 (-0.157, 0.027) | 0.168 |  |
| MCHC | Smokers | 0.048 (-0.152, 0.249) | 0.636 | 0.519 |  | Drinkers | 0.026 (-0.154, 0.206) | 0.774 | **0.002** |
|  | Non-smokers | -0.251 (-0.365, -0.138) | 1.53×10^-5^ |  |  | Non-drinkers | **-0.255 (-0.373, -0.138)** | **2.17×10^-5^** |  |
| RDW-CV | Smokers | 0.017 (-0.010, 0.044) | 0.223 | 0.117 |  | Drinkers | 0.025 (-0.001, 0.050) | 0.057 | 0.805 |
|  | Non-smokers | 0.019 (0.005, 0.033) | 0.009 |  |  | Non-drinkers | 0.016 (0.001, 0.030) | 0.034 |  |
| RDW-SD | Smokers | 0.035 (-0.033, 0.102) | 0.315 | 0.274 |  | Drinkers | 0.074 (0.018, 0.130) | 0.010 | 0.456 |
|  | Non-smokers | 0.055 (0.025, 0.086) | 4.08×10^-4^ |  |  | Non-drinkers | 0.043 (0.009, 0.076) | 0.012 |  |
| ^a^ GLMs with adjustment for smoking status, drinking status, work years, and workplace when appropriate.  ^b^ Adding an interaction term of BF% with smoking status and drinking status, respectively, in GLMs with adjustment for smoking status, drinking status, work years, workplace when appropriate. | | | | | | | | | |

| **Table S3.** Associations of plasma TC levels with erythroid-related hematologic parameters in different groups of gender, smoking status and drinking status. | | | | | | | | | | | | | | |
| --- | --- | --- | --- | --- | --- | --- | --- | --- | --- | --- | --- | --- | --- | --- |
| Erythroid-related hematologic parameters | Gender | β (95%CI) | *P*_value_^a^ | *P*_interaction_^b^ |  | Smoking status | β (95%CI) | *P*_value_^a^ | *P*_interaction_^b^ |  | Drinking status | β (95%CI) | *P*_value_^a^ | *P*_interaction_^b^ |
| RBC count | Female | 0.022 (-0.026, 0.070) | 0.369 | 0.457 |  | Smokers | -0.029 (-0.078, 0.020) | 0.244 | 0.113 |  | Drinkers | 0.023 (-0.024, 0.070) | 0.333 | 0.846 |
|  | Male | 0.000 (-0.033, 0.033) | 0.984 |  |  | Non-smokers | 0.028 (-0.005, 0.061) | 0.099 |  |  | Non-drinkers | -0.001 (-0.035, 0.033) | 0.944 |  |
| Hb | Female | **2.038 (0.663, 3.413)** | **0.004** | **0.032** |  | Smokers | 0.774 (-0.196, 1.744) | 0.119 | 0.136 |  | Drinkers | 0.979 (0.040, 1.919) | 0.042 | 0.068 |
|  | Male | **1.086 (0.422, 1.750)** | **1.00×10^-3^** |  |  | Non-smokers | 1.682 (0.918, 2.446) | 1.75×10^-5^ |  |  | Non-drinkers | 1.663 (0.886, 2.440) | 2.98×10^-5^ |  |
| HCT | Female | **0.627 (0.263, 0.992)** | **8.25×10^-4^** | **0.010** |  | Smokers | 0.147 (-0.114, 0.408) | 0.271 | **0.033** |  | Drinkers | **0.275 (0.029, 0.520)** | **0.029** | **0.027** |
|  | Male | **0.266 (0.087, 0.445)** | **0.004** |  |  | Non-smokers | **0.491 (0.287, 0.696)** | **2.75×10^-6^** |  |  | Non-drinkers | **0.448 (0.238, 0.657)** | **3.12×10^-5^** |  |
| MCV | Female | 0.795 (-0.155, 1.745) | 0.102 | 0.236 |  | Smokers | 0.717 (-0.035, 1.468) | 0.062 | 0.866 |  | Drinkers | 0.072 (-0.665, 0.808) | 0.849 | 0.064 |
|  | Male | 0.436 (-0.066, 0.939) | 0.089 |  |  | Non-smokers | 0.409 (-0.137, 0.954) | 0.142 |  |  | Non-drinkers | 0.833 (0.279, 1.387) | 0.003 |  |
| MCHC | Female | 0.601 (-0.766, 1.969) | 0.389 | 0.875 |  | Smokers | 0.319 (-0.520, 1.159) | 0.457 | 0.741 |  | Drinkers | 0.038 (-0.769, 0.844) | 0.927 | 0.853 |
|  | Male | 0.486 (-0.084, 1.057) | 0.095 |  |  | Non-smokers | 0.579 (-0.115, 1.274) | 0.103 |  |  | Non-drinkers | 0.713 (0.007, 1.418) | 0.048 |  |
| RDW-CV | Female | **-0.183 (-0.364, -0.003)** | **0.047** | **0.037** |  | Smokers | 0.063 (-0.054, 0.179) | 0.293 | **0.019** |  | Drinkers | 0.074 (-0.045, 0.192) | 0.222 | **0.028** |
|  | Male | -0.007 (-0.082, 0.068) | 0.852 |  |  | Non-smokers | **-0.116 (-0.206, -0.027)** | **0.011** |  |  | Non-drinkers | **-0.123 (-0.212, -0.034)** | **0.007** |  |
| RDW-SD | Female | 0.319 (-0.012, 0.650) | 0.060 | 0.892 |  | Smokers | **0.510 (0.242, 0.778)** | **2.12×10^-4^** | **0.030** |  | Drinkers | 0.278 (0.032, 0.525) | 0.027 | 0.894 |
|  | Male | 0.302 (0.129, 0.475) | 6.61×10^-4^ |  |  | Non-smokers | 0.158 (-0.025, 0.341) | 0.090 |  |  | Non-drinkers | 0.308 (0.114, 0.502) | 0.002 |  |
| ^a^ GLMs with adjustment for age, gender, smoking status, drinking status, work years, and workplace when appropriate.  ^b^ Adding an interaction term of TC and gender, smoking status, and drinking status, respectively, in GLMs with adjustment for age, gender, smoking status, drinking status, work years, workplace when appropriate. | | | | | | | | | | | | | | |

| **Table S4.** Associations of plasma TG levels with erythroid-related hematologic parameters in different groups of gender, smoking status and drinking status. | | | | | | | | | | | | | | |
| --- | --- | --- | --- | --- | --- | --- | --- | --- | --- | --- | --- | --- | --- | --- |
| Erythroid-related hematologic parameters | Gender | β (95%CI) | *P*_value_^a^ | *P*_interaction_^b^ |  | Smoking status | β (95%CI) | *P*_value_^a^ | *P*_interaction_^b^ |  | Drinking status | β (95%CI) | *P*_value_^a^ | *P*_interaction_^b^ |
| RBC count | Female | 0.021 (-0.020, 0.062) | 0.313 | 0.939 |  | Smokers | 0.030 (-0.003, 0.063) | 0.074 | 0.419 |  | Drinkers | 0.014 (-0.014, 0.041) | 0.331 | 0.259 |
|  | Male | 0.021 (-0.001, 0.043) | 0.061 |  |  | Non-smokers | 0.015 (-0.009, 0.038) | 0.222 |  |  | Non-drinkers | 0.030 (0.003, 0.058) | 0.031 |  |
| Hb | Female | 0.363 (-0.818, 1.544) | 0.547 | 0.612 |  | Smokers | 1.103 (0.452, 1.754) | 9.58×10^-4^ | 0.519 |  | Drinkers | 0.803 (0.256, 1.351) | 0.004 | 0.386 |
|  | Male | 0.989 (0.548, 1.430) | 1.22×10^-5^ |  |  | Non-smokers | 0.818 (0.270, 1.366) | 0.004 |  |  | Non-drinkers | 1.105 (0.471, 1.739) | 6.61×10^-4^ |  |
| HCT | Female | 0.013 (-0.297, 0.324) | 0.933 | 0.594 |  | Smokers | 0.231 (0.054, 0.408) | 0.011 | 0.465 |  | Drinkers | 0.138 (-0.005, 0.282) | 0.060 | 0.258 |
|  | Male | 0.190 (0.070, 0.309) | 0.002 |  |  | Non-smokers | 0.135 (-0.011, 0.281) | 0.070 |  |  | Non-drinkers | 0.223 (0.052, 0.394) | 0.011 |  |
| MCV | Female | -0.393 (-1.192, 0.406) | 0.335 | 0.612 |  | Smokers | -0.064 (-0.577, 0.448) | 0.806 | 0.826 |  | Drinkers | 0.003 (-0.427, 0.433) | 0.988 | 0.762 |
|  | Male | -0.011 (-0.346, 0.324) | 0.951 |  |  | Non-smokers | -0.038 (-0.425, 0.348) | 0.846 |  |  | Non-drinkers | -0.104 (-0.555, 0.347) | 0.651 |  |
| MCHC | Female | 0.983 (-0.161, 2.127) | 0.093 | 0.452 |  | Smokers | 0.476 (-0.094, 1.045) | 0.102 | 0.445 |  | Drinkers | 0.626 (0.158, 1.093) | 0.009 | 0.673 |
|  | Male | 0.602 (0.223, 0.980) | 0.002 |  |  | Non-smokers | 0.807 (0.317, 1.296) | 0.001 |  |  | Non-drinkers | 0.677 (0.105, 1.249) | 0.021 |  |
| RDW-CV | Female | 0.002 (-0.150, 0.155) | 0.977 | 0.947 |  | Smokers | 0.035 (-0.044, 0.114) | 0.390 | 0.131 |  | Drinkers | -0.004 (-0.073, 0.065) | 0.909 | 0.977 |
|  | Male | -0.011 (-0.061, 0.039) | 0.666 |  |  | Non-smokers | -0.042 (-0.106, 0.021) | 0.190 |  |  | Non-drinkers | -0.015 (-0.087, 0.057) | 0.686 |  |
| RDW-SD | Female | -0.112 (-0.407, 0.184) | 0.460 | 0.741 |  | Smokers | -0.008 (-0.193, 0.178) | 0.935 | 0.576 |  | Drinkers | -0.006 (-0.151, 0.138) | 0.934 | 0.474 |
|  | Male | -0.030 (-0.145, 0.085) | 0.611 |  |  | Non-smokers | -0.056 (-0.186, 0.073) | 0.393 |  |  | Non-drinkers | -0.075 (-0.236, 0.086) | 0.361 |  |
| ^a^ GLMs with adjustment for age, gender, smoking status, drinking status, work years, and workplace when appropriate.  ^b^ Adding an interaction term of TG and gender, smoking status, and drinking status, respectively, in GLMs with adjustment for age, gender, smoking status, drinking status, work years, workplace when appropriate. | | | | | | | | | | | | | | |

| **Table S5.** Associations of fatty liver occurrence with erythroid-related hematologic parameters in different groups of gender, smoking status and drinking status. | | | | | | | | | | | | | | |
| --- | --- | --- | --- | --- | --- | --- | --- | --- | --- | --- | --- | --- | --- | --- |
| Erythroid-related hematologic parameters | Gender | β (95%CI) | *P*_value_^a^ | *P*_interaction_^b^ |  | Smoking status | β (95%CI) | *P*_value_^a^ | *P*_interaction_^b^ |  | Drinking status | β (95%CI) | *P*_value_^a^ | *P*_interaction_^b^ |
| RBC count | Female | -0.078 (-0.216, 0.060) | 0.268 | 0.710 |  | Smokers | -0.114 (-0.215, -0.013) | 0.027 | 0.878 |  | Drinkers | -0.124 (-0.221, -0.027) | 0.013 | 0.701 |
|  | Male | -0.133 (-0.200, -0.066) | 1.00×10^-4^ |  |  | Non-smokers | -0.127 (-0.201, -0.053) | 7.89×10^-4^ |  |  | Non-drinkers | -0.124 (-0.199, -0.048) | 1.00×10^-3^ |  |
| Hb | Female | -3.594 (-7.474, 0.287) | 0.070 | 0.768 |  | Smokers | -3.259 (-5.265, -1.253) | 0.002 | 0.454 |  | Drinkers | -5.074 (-7.008, -3.140) | 3.73×10^-7^ | 0.199 |
|  | Male | -3.725 (-5.070, -2.379) | 6.96×10^-8^ |  |  | Non-smokers | -4.180 (-5.894, -2.465) | 2.01×10^-6^ |  |  | Non-drinkers | -2.889 (-4.630, -1.148) | 1.00×10^-3^ |  |
| HCT | Female | -1.216 (-2.229, -0.203) | 0.019 | 0.236 |  | Smokers | -0.737 (-1.279, -0.195) | 0.008 | 0.335 |  | Drinkers | -1.183 (-1.690, -0.677) | 5.70×10^-6^ | 0.559 |
|  | Male | -0.851 (-1.214, -0.487) | 5.06×10^-6^ |  |  | Non-smokers | -1.033 (-1.489, -0.577) | 9.79×10^-6^ |  |  | Non-drinkers | -0.722 (-1.189, -0.255) | 0.003 |  |
| MCV | Female | -0.906 (-3.513, 1.701) | 0.496 | 0.216 |  | Smokers | 0.645 (-0.938, 2.229) | 0.425 | 0.504 |  | Drinkers | -0.172 (-1.698, 1.355) | 0.826 | 0.390 |
|  | Male | 0.672 (-0.352, 1.695) | 0.199 |  |  | Non-smokers | -0.213 (-1.417, 0.991) | 0.729 |  |  | Non-drinkers | 0.819 (-0.412, 2.049) | 0.193 |  |
| MCHC | Female | 1.020 (-2.630, 4.670) | 0.584 | 0.154 |  | Smokers | -1.355 (-3.103, 0.393) | 0.129 | 0.595 |  | Drinkers | -1.803 (-3.468, -0.138) | 0.034 | 0.057 |
|  | Male | -1.264 (-2.423, -0.104) | 0.033 |  |  | Non-smokers | -0.794 (-2.316, 727) | 0.307 |  |  | Non-drinkers | -0.386 (-1.937, 1.165) | 0.626 |  |
| RDW-CV | Female | -0.144 (-0.623, 0.334) | 0.555 | 0.542 |  | Smokers | -0.018 (-0.259, 0.223) | 0.883 | 0.775 |  | Drinkers | 0.031 (-0.209, 0.271) | 0.800 | 0.266 |
|  | Male | -0.031 (-0.181, 0.119) | 0.686 |  |  | Non-smokers | -0.061 (-0.254, 0.132) | 0.537 |  |  | Non-drinkers | -0.104 (-0.297, 0.089) | 0.290 |  |
| RDW-SD | Female | **-1.036 (-1.937, -0.136)** | **0.025** | **0.022** |  | Smokers | 0.166 (-0.409, 0.741) | 0.572 | 0.335 |  | Drinkers | 0.066 (-0.460, 0.593) | 0.805 | 0.598 |
|  | Male | 0.119 (-0.239, 0.477) | 0.514 |  |  | Non-smokers | -0.216 (-0.622, 0.190) | 0.297 |  |  | Non-drinkers | -0.134 (-0.563, 0.296) | 0.543 |  |
| ^a^ GLMs with adjustment for age, gender, smoking status, drinking status, work years, and workplace when appropriate.  ^b^ Adding an interaction term of the occurrence of fatty liver and gender, smoking status, and drinking status, respectively, in GLMs with adjustment for age, gender, smoking status, drinking status, work years, workplace when appropriate. | | | | | | | | | | | | | | |

| **Table S6.** Associations of urinary SPMA with erythroid-related hematologic parameters in different fat content categories. | | | | | | | | | | | | | | | |
| --- | --- | --- | --- | --- | --- | --- | --- | --- | --- | --- | --- | --- | --- | --- | --- |
| Fat content categories | RBC count | | |  | Hb | | |  | HCT | | |  | MCV | | |
|  | β (95% CI) | *P*_trend_^a^ | *P*_interaction_ ^b^ |  | β (95% CI) | *P*_trend_^a^ | *P*_interaction_ ^b^ |  | β (95% CI) | *P*_trend_^a^ | *P*_interaction_ ^b^ |  | β (95% CI) | *P*_trend_^a^ | *P*_interaction_ ^b^ |
| **BF%** |  |  |  |  |  |  |  |  |  |  |  |  |  |  |  |
| Low | -0.007 (-0.041, 0.026) | 0.666 | 0.127 |  | -0.088 (-0.769, 0.593) | 0.800 | 0.148 |  | -0.181 (-0.365, 0.003) | 0.054 | 0.145 |  | -0.198 (-0.713, 0.316) | 0.450 | 0.492 |
| Median | -0.012 (-0.048, 0.024) | 0.502 |  |  | -0.490 (-1.230, 0.250) | 0.194 |  |  | -0.166 (-0.368, 0.036) | 0.107 |  |  | -0.208 (-0.764, 0.347) | 0.462 |  |
| High | -0.008 (-0.043, 0.027) | 0.658 |  |  | 0.087 (-0.804, 0.979) | 0.848 |  |  | -0.107 (-0.340, 0.126) | 0.367 |  |  | -0.161 (-0.787, 0.465) | 0.614 |  |
| **TC** |  |  |  |  |  |  |  |  |  |  |  |  |  |  |  |
| Low | -0.002 (-0.039, 0.036) | 0.937 | 0.934 |  | -0.728 (-1.575, 0.119) | 0.092 | 0.580 |  | -0.301 (-0.526, -0.076) | 0.009 | 0.580 |  | -0.612 (-1.238, 0.014) | 0.055 | 0.552 |
| Median | -0.009 (-0.044, 0.026) | 0.617 |  |  | -0.132 (-0.876, 0.612) | 0.728 |  |  | -0.188 (-0.384, 0.009) | 0.061 |  |  | -0.225 (-0.777, 0.327) | 0.424 |  |
| High | -0.021 (-0.054, 0.013) | 0.230 |  |  | 0.058 (-0.679, 0.796) | 0.876 |  |  | -0.079 (-0.282, 0.124) | 0.444 |  |  | 0.162 (-0.370, 0.694) | 0.551 |  |
| **TG** |  |  |  |  |  |  |  |  |  |  |  |  |  |  |  |
| Low | -0.019 (-0.055, 0.018) | 0.312 | 0.190 |  | -0.702 (-1.506, 0.102) | 0.087 | 0.260 |  | **-0.316 (****-0.536, -0.095)** | **0.005** | **0.050** |  | -0.437 (-1.065, 0.191) | 0.173 | 0.603 |
| Median | -0.008 (-0.043, 0.026) | 0.635 |  |  | 0.414 (-0.359, 1.186) | 0.294 |  |  | -0.043 (-0.251, 0.165) | 0.685 |  |  | 0.072 (-0.479, 0.622) | 0.799 |  |
| High | -0.004 (-0.039, 0.032) | 0.842 |  |  | -0.486 (-1.229, 0.256) | 0.199 |  |  | **-0.202 (****-0.396,** **-0.007)** | **0.042** |  |  | -0.292 (-0.838, 0.253) | 0.294 |  |
| **Fat liver** |  |  |  |  |  |  |  |  |  |  |  |  |  |  |  |
| No | -0.003 (-0.026, 0.020) | 0.810 | 0.224 |  | -0.210 (-0.726, 0.306) | 0.425 | 0.512 |  | -0.029 (-0.075, 0.018) | 0.230 | 0.878 |  | -0.363 (-0.736, 0.010) | 0.057 | 0.189 |
| Yes | -0.043 (-0.083, -0.004) | 0.031 |  |  | -0.457 (-1.252, 0.338) | 0.260 |  |  | 0.027 (-0.051, 0.105) | 0.497 |  |  | 0.344 (-0.279, 0.967) | 0.280 |  |
| ^a^ Covariate-adjusted GLMs.  ^b^ *P*_interaction_ was calculated by modeling an interaction term of SPMA and fat content categories in covariate-adjusted GLMs. | | | | | | | | | | | | | | | |

| **Table S6 (Continued).** Associations of urinary SPMA with erythroid-related hematologic parameters in different fat content categories. | | | | | | | | | | | |
| --- | --- | --- | --- | --- | --- | --- | --- | --- | --- | --- | --- |
| Fat content categories | MCHC | | |  | RDW-CV | | |  | RDW-SD | | |
|  | β (95% CI) | *P*_trend_^a^ | *P*_interaction_ ^b^ |  | β (95% CI) | *P*_trend_^a^ | *P*_interaction_ ^b^ |  | β (95% CI) | *P*_trend_^a^ | *P*_interaction_ ^b^ |
| **BF%** |  |  |  |  |  |  |  |  |  |  |  |
| Low | **1.170 (****0.558,** **1.782)** | **1.78×10^-4^** | **0.021** |  | 0.018 (-0.061, 0.097) | 0.660 | 0.326 |  | 0.050 (-0.128, 0.229) | 0.580 | 0.408 |
| Median | 0.279 (-0.335, 0.894) | 0.373 |  |  | 0.017 (-0.058, 0.092) | 0.661 |  |  | -0.013 (-0.217, 0.191) | 0.902 |  |
| High | **1.021 (****0.222,** **1.819)** | **0.012** |  |  | -0.093 (-0.204, 0.018) | 0.100 |  |  | -0.100 (-0.324, 0.124) | 0.383 |  |
| **TC** |  |  |  |  |  |  |  |  |  |  |  |
| Low | 0.758 (-0.007, 1.523) | 0.052 | 0.460 |  | 0.029 (-0.071, 0.130) | 0.568 | 0.682 |  | -0.109 (-0.321, 0.103) | 0.313 | 0.689 |
| Median | 1.367 (0.712, 2.022) | 4.31×10^-5^ |  |  | -0.023 (-0.114, 0.068) | 0.621 |  |  | -0.035 (-0.232, 0.162) | 0.728 |  |
| High | 0.720 (0.067, 1.373) | 0.031 |  |  | -0.073 (-0.158, 0.012) | 0.092 |  |  | 0.037 (-0.163, 0.237) | 0.716 |  |
| **TG** |  |  |  |  |  |  |  |  |  |  |  |
| Low | 1.110 (0.358, 1.863) | 0.004 | 0.264 |  | -0.028 (-0.131, 0.075) | 0.594 | 0.856 |  | -0.018 (-0.235, 0.200) | 0.873 | 0.648 |
| Median | 1.327 (0.646, 2.009) | 1.34×10^-4^ |  |  | -0.032 (-0.122, 0.058) | 0.483 |  |  | 0.032 (-0.167, 0.230) | 0.753 |  |
| High | 0.435 (-0.214, 1.084) | 0.189 |  |  | -0.008 (-0.092, 0.076) | 0.854 |  |  | -0.084 (-0.284, 0.116) | 0.412 |  |
| **Fatty liver** |  |  |  |  |  |  |  |  |  |  |  |
| No | 0.105 (-0.047, 0.258) | 0.177 | 0.137 |  | 0.000 (-0.021, 0.020) | 0.966 | 0.960 |  | -0.001 (-0.045, 0.043) | 0.969 | 0.228 |
| Yes | -0.131 (-0.399, 0.136) | 0.336 |  |  | 0.015 (-0.014, 0.045) | 0.316 |  |  | 0.007 (-0.077, 0.092) | 0.863 |  |
| ^a^ Covariate-adjusted GLMs.  ^b^ *P*_interaction_ was calculated by modeling an interaction term of SPMA and fat content categories in covariate-adjusted GLMs. | | | | | | | | | | | |
